# Bacteria and parasites in *Podarcis sicula* and *P. sicula klemmerii*

**DOI:** 10.1186/s12917-018-1708-5

**Published:** 2018-12-10

**Authors:** Ludovico Dipineto, Pasquale Raia, Lorena Varriale, Luca Borrelli, Vittorio Botta, Carmela Serio, Michele Capasso, Laura Rinaldi

**Affiliations:** 1 Dipartimento di Medicina Veterinaria e Produzioni Animali, Università di Napoli Federico II, Naples, Italy; 2 Dipartimento di Scienze della Terra, dell’Ambiente e delle Risorse, Università di Napoli Federico II, Naples, Italy

**Keywords:** *Podarcis sicula*, Italian wall lizard, Bacteria, Parasites, FLOTAC

## Abstract

**Background:**

New epidemiological data on bacterial and parasitic infections in 24 Italian wall lizards, namely *Podarcis sicula* (mainland population) and *P. sicula klemmerii* (insular population) in southern Italy were provided. To achieve this goal, samples were collected from individuals belonging to the two populations and analysed by microbiological and parasitological methods.

**Results:**

A wide range of bacteria (e.g. *Pantoea* spp., *Citrobacter* spp., *Morganella* spp., *Pseudomonas*, *Enterobacter* spp., *Staphylococcus* spp. and *Escherichia coli*) and parasites (e.g. *Ophionyssus natricis*, coccidia, Dicrocoelidae) were detected in both *P. sicula* and *P. sicula klemmerii* individuals. Insular population presented similar bacterial and parasitic diversity to its mainland counterpart. Ampicillin was the antimicrobial with the highest resistance rate.

**Conclusion:**

This study highlighted various bacteria and parasites, some of them potentially zoonotic. Further studies are needed to better understand the epidemiology and transmission routes of these pathogens along with their impact on the welfare and behaviour of Italian wall lizards.

## Background

The Italian wall lizard, *Podarcis sicula*, is one of the most common lizards in Italy [1]. The snout-vent length (SVL) is, on average, 15 to 25 cm long. The colour pattern is characterized by a green or brown back and whitish belly, although melanic variants, with either shorter, or longer SVL, are known to occur in several islands in the Mediterranrean sea. As a common, and easily managed study model, the Italian wall lizard was subjected to several studies, regarding aspects as disparate as phenotypic response to predation [2], feeding behaviour [3], ontogeny [4], adaptation to novel environments [5], biogeography [6, 7], and its role as a biological indicator [8].

*Podarcis sicula klemmeri* [9] is one of the subspecies belonging to *P. sicula*. It is confined to a small, 1 km^2^ large islet, Licosa, off the western coast of Italy. As with many other insular populations of the Italian wall lizard, *P.s. klemmeri* is melanic, meaning the back appears tinged with blue, and the pale underside is bluish as well, rather than the usual white of continental populations.

Melanic variants have been investigated for the so-called Island syndrome [10], which is a suite of phenotypic character shifts in insular populations including, besides melanism, changed body size, feeding behaviour and ecology, patterns of aggressiveness, and life history traits [11–15]. The link between melanism and characters shifts on islands was found to be in the activity levels of melano-cortin receptors, MCRs [13]. Melanocortins form a suite of five receptors activated pleyotropically by a single DNA locus, the proopiomelanocortin POMC gene, that happen to regulate feeding and sexual activity, immunocompetence and body colour [16–18]. Interestingly, Monti et al. [19] tested whether *P. s. klemmeri* individuals have different ectoparasite loads (i.e. the density of ticks and mites on the skin) as compared to the continental individuals. They found reduced load in insular individuals, consistently with their comparatively higher α-melanocyte-stimulating hormone (MSH) levels.

Herein, we deepen microbiological and parasitological investigations on *P. s. klemmeri*. Besides a few studies [20, 21], these aspects have not been scrutinized so far, yet remain very important in the case of melanic insular lizards, whose immune system is expected to be depressed by great investment in reproduction, and life at high density [13, 19, 22]. This study was undertaken with the aim to evaluate the presence of potentially zoonotic bacteria and parasites in wild-caught insular individuals of *P. sicula klemmeri*, along with mainland individuals of *P. sicula*.

## Results

A wide range of bacteria and parasites were detected in both *P. sicula* and *P. sicula klemmerii* individuals. Among the 24 analysed animals, 23 (95.9%) were positive to at least one bacterium and 19 (79.1%) were positive to at least one parasite.

Regarding microbiological analysis, *Pantoea* spp. was isolated in 4/24 (16.7%) oral swabs, *Citrobacter* spp. in 1/24 (4.2%), *Morganella morganii* in 1/24 (4.2%), *Pseudomonas aeruginosa* in 1/24 (4.2%) and Coagulase-Negative Staphylococci (NCS) in 7/24 (29.1%). For cloacal swabs, *Citrobacter* spp. was found in 10/24 (41.7%) animals of which 1/10 (10.0%) was identified as *Citrobacter koseri*, *Enterobacter* spp.was found in 8/24 (33.3%) animals of which 1/8 (12.5%) was identified as *Enterobacter aerogenes*, *Escherichia coli* was found in 3/24 (12.5%) animals of which 1/3 (33.3%) was serotyped as serogroup O 145, *Morganella morganii* was isolated in 2/24 (8.3%) individuals, S*hewanella* spp. in 1/24 (4.2%), *Providencia* spp. in 2/24 (8.3%), Coagulase-Negative Staphylococci (NCS) in 20/24 (83.3%) and *Pseudomonas* spp. in 10/24 (41.7%) individuals. Several bacteria were routinely isolated from the same animal.

With respect to antimicrobial susceptibility testing, *Citrobacter* spp. showed the highest resistance profile. Specifically, two out of ten strains of *Citrobacter* spp. were resistant to three or more drugs (“multidrug-resistant”) of which, one strain was resistant to ampicillin, doxycycline and streptomycin and the other one was resistant to ampicillin, amoxicillin-clavulanic acid, doxycycline and nitrofurantoin. The remaining strains were resistant to ampicillin, one was also resistant to doxycycline and one other was also resistant to amoxicillin-clavulanic acid. *Escherichia coli* O145 and all the strains of *Enterobacter* spp. were resistant to ampicillin. In contrast, *Pseudomonas aeruginosa* were susceptible to all antimicrobials tested.

Regarding the parasitological results, the scotch test highlighted the presence of *Ophionyssus natricis* mite. During the faecal examination, eggs of pinworms (20/24; 83.3%), *O. natricis* (12/24; 50%), and Dicrocoelidae (6/24; 25%) as well as oocysts of coccidia (11/24; 45.8%) were detected. As with bacteria, different parasite species were simultaneously detected from the same animal. In addition, adult liver flukes (Dicrocoelidae) were also found during the necropsy. The parasitological and microbiological results related to each lizard are detailed in Table 1.

**Table 1:** Parasites and bacteria found in *Podarcis sicula klemmeri* (LIC) and *P. sicula* (PLIC) lizards

Lizard ID	Parasites				Bacteria	
		EPG/OPG ^a^			Swab samples	
	Parasite	NaCl	ZnSO_4_	*O. natricis*	Cloacal	Oral
LIC 1001	Negative	–	–	Positive	*Enterobacter* spp.*, Staphylococcus* spp.*, Pseudomonas* spp.	*Pantoea* spp.*, Staphylococcus* spp.
LIC 1002	Pinworms Coccidae	300 405	225 270	Positive	*Enterobacter aerogenes, Citrobacter* spp.*, Staphylococcus* spp.	*Staphylococcus* spp.
LIC 1003	Pinworms Coccidae	720 120	150	Positive	*Morganella morganii, Citrobacter* spp.*, Staphylococcus* spp.	*Pantoea* spp.
LIC 1004	Pinworms Coccidae	720 120	150	Positive	*Citrobacter* spp.*, Shewanella* spp.*, E. coli, Staphylococcus* spp.*, Pseudomonas* spp.	*Pantoea* spp.*, Staphylococcus* spp.
LIC 1005	Pinworms Liver flukes	2.880	1.440 960	Positive	*Citrobacter* spp.*, Staphylococcus* spp.	*Staphylococcus* spp.
LIC 1006	Pinworms Liver flukes	2.880 120	1.440 120	Positive	*Morganella morganii, Citrobacter* spp.*, Pseudomonas* spp.	*Morganella morganii, Citrobacter* spp.*, Pantoea* spp. *Pantoea,PStaphylococcus*
LIC 1007	Pinworms Coccidae	3.600 4.140	2.100 1.530	Positive	*Enterobacter* spp.*, E. coli, Staphylococcus* spp.	–
LIC 1008	Negative	–	–	Positive	*Enterobacter* spp.*, Providencia* spp.*, Staphylococcus* spp.	–
LIC 1009	Pinworms Coccidae	3.600 4.140	2.100 1.530	Positive	*E. coli* O145, *Staphylococcus* spp.	–
LIC 1010	Pinworms	330	300	Positive	*Staphylococcus* spp.	–
LIC 1011	Pinworms Liver flukes	3.420	1.890 120	Positive	*Pseudomonas* spp.	–
LIC 1012	Pinworms	3.420	1.890	Positive	*Citrobacter* spp.*, Providencia* spp.*, Staphylococcus* spp.*, Pseudomonas* spp.	*Pseudomonas aeruginosa, Staphylococcus* spp.
LIC 1013	Pinworms Coccidae	870 360	690 105	–	*Enterobacter* spp.*, Staphylococcus* spp.	–
LIC 1014	Pinworms Coccidae	870 360	690 105	–	*Citrobacter koseri, Staphylococcus* spp.*, Pseudomonas* spp.	–
LIC 1015	Pinworms Liver flukes	540	300 7800	–	*Enterobacter* spp.*, Staphylococcus* spp.	–
LIC 1016	Pinworms Liver flukes	540	300 42	–	*Staphylococcus* spp.*, Pseudomonas* spp.	–
LIC 1017	Pinworms	540	300	–	*Pseudomonas* spp.	–
LIC 1018	Pinworms Coccidae	360 90	450	–	*Staphylococcus* spp.	–
LIC 1019	Pinworms Coccidae Liver flukes	360 90	450 48	–	*Staphylococcus* spp.*, Pseudomonas* spp.	–
LIC 1020	Pinworms Coccidae	360 90	450 48	–	*Enterobacter* spp.*, Staphylococcus* spp.	–
PLIC 1001	Pinworms	420	375	–	NOT IDENTIFIED	*Staphylococcus* spp.
PLIC 1002	Negative	–	–	–	*Enterobacter* spp.*, Citrobacter* spp.*, Staphylococcus* spp.	–
PLIC 1003	Negative	–	–	–	*Citrobacter* spp.*, Staphylococcus* spp.	*Staphylococcus* spp.
PLIC 1004	Negative	–	–	–	*Citrobacter* spp.*, Staphylococcus* spp.*, Pseudomonas* spp.	–

^a^EPG/OPG = eggs/oocysts per gram of faeces

## Discussion

Our results provide new epidemiological data on bacterial and parasitic infections in *P. sicula* and *P. sicula klemmerii*. These species were poorly investigated from a sanitary perspective so far. Bacterial and parasitic infections in reptiles have recently gained scientific relevance. However, the majority of studies were carried out on captive-bred individuals of Cryptodira (tortoises, [23]), Serpentes and Sauria (snakes and other ‘lizards’, [24]). Conversely, data on infections in wild lizards are scarce.

The results of our study showed the presence of endoparasites (coccidia, pinworms and liver flukes) and ectoparasites (*O. natricis*) in *P. sicula* and *P. sicula klemmeri.* With respect to the presence of pinworms, our findings are in line with those by Casanova et al. [20] who detected the presence of a nematode belonging to the Oxyuroidea superfamily in *P. sicula*. Nevertheless*,* our results showed the first epidemiological information on parasites infecting *P. sicula klemmeri*. Regarding the presence of *O. natricis,* there are no studies in literature that highlight its presence in *P. sicula* and *P. sicula klemmeri,* although the infestation by this mite has been described in various genus of Sauria as *Lacerta*, *Podarcis* and *Darevskia*. In addition, the carrier role of *O. natricis* in the transmission of a blood parasite belonging to the genus *Karyolysus* has been reported in lizards [25]. An interesting finding of our study was the detection at necropsy of liver flukes (Dicrocoelidae) in the continental lizards, although species identification was not performed.

Bacteriological results also added new epidemiological data in *P. sicula* due to detection of some potential zoonotic species as *P. aeruginosa* and *E. coli* O145. The bacterial isolation performed on oral swabs gave as result the presence of bacteria belonging to the genera *Pantoea, Pseudomonas, Morganella, Staphylococcus, Citrobacter, Shewanella* never detected before in *P. sicula* and *P. sicula klemmeri*. However, these bacteria are frequent in reptiles, along with other bacteria such as *Enterobacter* spp*., E. coli* and *Providencia* spp. [26–28]. Bacteria isolated from cloacal swabs were *Enterobacter* spp*., Citrobacter* spp*., Morganella* spp*., E. coli, Providencia* spp*. Shewanella* spp., and *Pseudomonas* spp., gram-negative bacteria frequently isolated in other reptiles [29] as well as *Staphylococcus* spp. The isolation of *E. coli* O145 in one *P. sicula klemmeri* is noteworthy due to the potential zoonotic role of this serogroup which is considered a shigatoxin-producing *E. coli*.

It is interesting to notice that the insular population presents similar bacterial diversity of its mainland counterpart, although the differences in sample size urge caution in interpreting these results. Noteworthy, 9 different bacterial genera were identified in *P.s. klemmeri* (up to six in a single individual), against 4 in mainland individuals (up to three in a single individual). A weakness of this study was the failure to isolate bacteria in the oral cavity of some lizards due to the difficult to keep viable some strains during the isolation procedures. To our knowledge this is the first study to assess the antimicrobial resistance profiles of potentially zoonotic bacteria carried by *Podarcis* spp. It is difficult to speculate regarding the results of the antimicrobial resistance recovered in the present study. However, one possible mechanism by which lizards acquire antimicrobial resistant bacteria in their environment range may be directly through exposure to human or livestock waste, or indirectly through consumption of prey which may harbor resistant bacteria.

The figure for parasites is even harder to interpret given the smaller sample size. Yet, we found coccidia, liver flukes and pinworms in the insular populations, and pinworms only (in just one individual) within continental lizards. While difference in collecting season, sample size, and the instance of necropsy in two individuals only suggest great caution, data seem to indicate an overall higher parasite/bacterial load in insular lizards. Assuming this is true, it remains to be elucidated whether this depends on population density on Licosa, or on less competent immune system in insular melanic individuals [12, 13].

## Conclusion

In conclusion, the results of this study highlighted various bacteria and parasites, some of them pathogenic, able to infect the species *P. sicula* and the subspecies *P. sicula klemmeri*. Further studies are needed to better understand the epidemiology and transmission routes of these pathogens along with their impact on the welfare and behaviour of Italian wall lizards.

## Methods

### Study animals

From November 2015 to May 2016, we collected and examined, within 24 h from capture, a total of 24 individuals, 20 (14 males and 10 females) belonging to the subspecies *P. sicula klemmeri*, and 4 individuals belonging to the species *P. sicula*. The *P. sicula klemmeri* specimens were from the small islet of Licosa, whereas the *P. sicula* specimens from Punta Licosa mainland (Campania region of southern Italy). The study site, collection and housing methods were described in Raia et al. [12, 13]. Lizards captured on Licosa islet were identified with the code “LIC” and a progressive number (from LIC 1001 to 1020); the lizards from Licosa mainland were identified with the code “PLIC” and a progressive number (from PLIC 1001 to 1004).

In order to perform the microbiological analysis, oral and cloacal samples were individually collected by sterile cotton-tipped swabs and then inoculated in Phosphate Buffered Saline (PBS). For the parasitological analyses, different methods were used: faecal samples were collected from each lizard, scotch tape test was performed on the skin of each animal and, eventually, a necropsy with stereo microscope was carried out on two lizards found dead on the insular study site. After sampling, animals were released in the same area where they were captured. All experiments described were performed in accordance with the local and national guidelines governing animal experiments (86/ 609/CEE and its modifications).

### Microbiological and parasitological analyses

Oral and cloacal swabs stored in PBS were inoculated in buffered peptone water (BPW), *Campylobacter*-selective enrichment broth (CSEB), cooked meat medium (CMM), modified tryptone soy broth (MTSB). The samples inoculated in BPW were incubated at both 25 °C and 37 °C for 24 h and then inoculated/streaked into Rappaport-Vassiliadis broth (RV), Columbia blood agar base (CBA; Oxoid, Milan, Italy), *Pseudomonas* cetrimide agar (PCA; Oxoid), MacConkey agar (MCA; Oxoid) and Baird-Parker agar (BPA; Oxoid). The samples inoculated in MTSB were incubated at both 25 °C and 37 °C for 24 h and then spread on sorbitol MacConkey agar (SMCA; Oxoid). The samples inoculated in CSEB were incubated in microaerophilic atmosphere at both 25 °C and 42 °C for 48 h and then streaked on *Campylobacter* blood-free selective agar (CBFA; Oxoid). Instead, the samples inoculated in CMM were incubated in anaerobic atmosphere at both 25 °C and 37 °C for 24 h, and then streaked on anaerobe basal agar (ABA; Oxoid). The remaining PBS were incubated at 4 °C for 14 days, spread on *Yersinia* selective agar base (cefsulodin-irgasan-novobiocin, CIN Agar; Oxoid) and incubated at 30 °C for additional 24–48 h. CBA, PCA, MCA, SMCA, CEOA and BPA were incubated at both 25 °C and 37 °C for 24–48 h, whereas RV was incubated at both 25 °C and 42 °C for 24–48 h and then streaked on xylose lysine desoxycholate agar (XLD) and brilliant green agar (BGA); CBFA were incubated in microaerophilic atmosphere at both 25 °C and 42 °C for 24–48 h, ABA plates were anaerobically incubated both at 25 °C and 37 °C for 48 h and checked daily for a further week before discarding. All the isolates were previously identified according to their morphological features, their growing need, Gram colouring, motility and pigments production test and through conventional biochemical and phenotypic test. Progressively, the isolates were identified through the biochemical systems API 20 E, API 20 NE (bioMerieux, Mercy-l’Etoile, France) and RapID ANA II, RapID NF PLUS, RapID STAPH PLUS (Oxoid). *Escherichia coli* strains were serogrouped with antisera poly- and monospecific (Sifin) whereas, suspected *Campylobacter* strains were identified by PCR as reported by Gargiulo et al. [30].

In order to detect and count parasitic elements (eggs, larvae, cysts, oocysts), each faecal sample collected from the 24 lizards was analysed with the FLOTAC pellet technique [24, 31]. The analytic sensitivity of the FLOTAC pellet technique varied on the basis of the weight of each pellet μμ [24] and expressed as eggs/oocysts per gram (EPG/OPG) of faeces. Two flotation solutions were used: sodium chloride (NaCl, specific gravity = 1200) and zinc sulfate (ZnSO4, specific gravity = 1350) to detect protozoa/nematoda and trematoda, respectively. The scotch tape test was performed on the skin of the lizards, specifically, at the level of skin folds, around the eye, the cloaca and the eardrum recesses. The necropsy of the two lizard carcasses was performed under a stereomicroscope using the reptile necropsy protocol reported in Jacobson [32].

### Antimicrobial susceptibility tests

*Pseudomonas aeruginosa*, *Escherichia coli* O145, *Citrobacter* spp., *Enterobacter* spp. isolates were submitted to antimicrobial susceptibility testing using the disc diffusion method according to Clinical Laboratory Standard Institute [33]. The antimicrobials tested were ampicillin (10 μg), ceftazidime (30 μg), ciprofloxacin (5 μg), enrofloxacin (5 μg), sulphamethoxazole-trimethoprim (25 μg), nalidixic acid (30 μg), amoxicillin-clavulanic acid (30 μg), doxycycline (30 μg), gentamicin (10 μg), streptomycin (10 μg), amikacin (30 μg), nitrofurantoin (30 μg), colistin sulphate (10 μg), piperacillin 100 (μg) and Cefpodoxime Combination Disc Kit (Oxoid). The inhibition zones were measured and scored as sensitive, intermediate susceptibility and resistant according to CLSI documents [34].

## Abbreviations

ABA: Anaerobe basal agar
BGA: Brilliant green agar
BPA: Baird-Parker agar
BPW: Buffered peptone water
CBA: Columbia blood agar base
CBFA: *Campylobacter* blood-free selective agar
CIN: Cefsulodin-irgasan-novobiocin
CMM: Cooked meat medium
CSEB: *Campylobacter*-selective enrichment broth
EPG/OPG: eggs/oocysts per gram
MCA: MacConkey agar
MCRs: Melano-cortin receptors
MSH: α-melanocyte-stimulating hormone
MTSB: Modified tryptone soy broth
NaCl: Sodium chloride
PBS: Phosphate Buffered Saline
PCA: *Pseudomonas* cetrimide agar
PCR: Polymerase chain reaction
RV: Rappaport-Vassiliadis broth
SMCA: Sorbitol MacConkey agar
SVL: Snout-vent length
XLD: Xylose lysine desoxycholate agar
ZnSO4: Zinc sulphate
